# A Dual Architecture Fusion and AutoEncoder for Automatic Morphological Classification of Human Sperm

**DOI:** 10.3390/s23146613

**Published:** 2023-07-22

**Authors:** Muhammad Izzuddin Mahali, Jenq-Shiou Leu, Jeremie Theddy Darmawan, Cries Avian, Nabil Bachroin, Setya Widyawan Prakosa, Muhamad Faisal, Nur Achmad Sulistyo Putro

**Affiliations:** 1 Department of Electronic and Computer Engineering, National Taiwan University of Science and Technology, Taipei City 10607, Taiwan or izzudin@uny.ac.id (M.I.M.); jeremie.darmawan@student.i3l.ac.id (J.T.D.); d10902810@mail.ntust.edu.tw (C.A.); d10702804@mail.ntust.edu.tw (S.W.P.); d10802803@mail.ntust.edu.tw (M.F.); or nur.achmad.s.p@ugm.ac.id (N.A.S.P.); 2 Department of Electronic and Informatic Engineering Education, Universitas Negeri Yogyakarta, Yogyakarta 55281, Indonesia; 3 Department of Bioinformatics, Indonesia International Institute for Life Science, Jakarta 13210, Indonesia; 4 Departement of Electrical Engineering, National Taiwan University of Science and Technology, Taipei City 10607, Taiwan; m11107814@mail.ntust.edu.tw; 5 Department of Computer Science and Electronics, Universitas Gadjah Mada, Yogyakarta 55281, Indonesia

**Keywords:** deep learning, dual architecture fusion, morphological classification, sperm, swin transformer

## Abstract

Infertility has become a common problem in global health, and unsurprisingly, many couples need medical assistance to achieve reproduction. Many human behaviors can lead to infertility, which is none other than unhealthy sperm. The important thing is that assisted reproductive techniques require selecting healthy sperm. Hence, machine learning algorithms are presented as the subject of this research to effectively modernize and make accurate standards and decisions in classifying sperm. In this study, we developed a deep learning fusion architecture called SwinMobile that combines the Shifted Windows Vision Transformer (Swin) and MobileNetV3 into a unified feature space and classifies sperm from impurities in the SVIA Subset-C. Swin Transformer provides long-range feature extraction, while MobileNetV3 is responsible for extracting local features. We also explored incorporating an autoencoder into the architecture for an automatic noise-removing model. Our model was tested on SVIA, HuSHem, and SMIDS. Comparison to the state-of-the-art models was based on F1-score and accuracy. Our deep learning results accurately classified sperm and performed well in direct comparisons with previous approaches despite the datasets’ different characteristics. We compared the model from Xception on the SVIA dataset, the MC-HSH model on the HuSHem dataset, and Ilhan et al.’s model on the SMIDS dataset and the astonishing results given by our model. The proposed model, especially SwinMobile-AE, has strong classification capabilities that enable it to function with high classification results on three different datasets. We propose that our deep learning approach to sperm classification is suitable for modernizing the clinical world. Our work leverages the potential of artificial intelligence technologies to rival humans in terms of accuracy, reliability, and speed of analysis. The SwinMobile-AE method we provide can achieve better results than state-of-the-art, even for three different datasets. Our results were benchmarked by comparisons with three datasets, which included SVIA, HuSHem, and SMIDS, respectively (95.4% vs. 94.9%), (97.6% vs. 95.7%), and (91.7% vs. 90.9%). Thus, the proposed model can realize technological advances in classifying sperm morphology based on the evidential results with three different datasets, each having its characteristics related to data size, number of classes, and color space.

## 1. Introduction

### 1.1. Main Problem

Reproduction is a cornerstone of life that aims to secure the prolongation of hereditary features or the gene pool [1]. Problems in this aspect of life are associated with social, cultural, and medical issues [2]. The problems can be described as extensive and comprehensive, and many variables should be considered when looking for a solution. One of the frequent problems in reproduction in recent years is infertility. This issue can be further categorized into two sub-categories: sub-fertility and infertility [3]. Sub-fertility and infertility are closely related. However, the differences may be attributed to the duration of unwanted non-conception. Sub-fertility is defined as the prolonged duration of non-conception, while infertility can be considered sterility with sporadic conception moments. As mentioned, reproductive issues encompass many aspects of life, and choices in lifestyle have a significant role in fertility [4]. Some lifestyle choices might negatively influence fertility, especially when made excessively. 

Fat-rich diets, the use of recreational drugs, sexual activity, smoking, alcohol misuse, and mental conditions such as anxiety, stress, and depression are only some examples of lifestyle choices that promote infertility. With that said, around 10% of couples experience infertility [5]. Delayed childbearing also drastically reduces the probability of conceiving [4,5]. To circumvent these problems and promote conception, assisted reproduction techniques (ART), such as IVF and ICSI, have been developed and are available [5]. IVF stands for in vitro fertilization, while ICSI is the abbreviation for intracytoplasmic sperm injection [6]. The assisted reproduction technique unites gametes in vitro and bypasses the process of sexual intercourse [5]. The resulting embryo can be stored, exchanged, designed, altered, and implanted in any womb. This method may be significant, as it is independent of sexual orientation, age, and gender.

### 1.2. Specific Problem

For the ART process to be lucrative, quality sperm need to be selected before being inserted into the ova [7]. However, a universal parameter that defines the quality of sperm has not been developed, and selection has been made subjectively based on qualitative assessment. The ideal method for sperm selection should have two properties: namely, it should be non-invasive and cost-effective. One of the solutions is CASA, an acronym for computer-aided sperm analysis, which refers to the multi-image system of analyzing and extracting objective information related to sperm motion or morphology using computers. These systems project sperm images onto a detector, which will detect objects based on the pixel’s light intensity, extract desired information, and output them [8]. This is similar to the principle behind computer vision models that use pixel brightness, color, and texture similarity to make inferences [9]. CASA has been used extensively in clinical laboratories and hospitals worldwide for semen analysis procedures [10].

There has been difficulty in applying this technology in human sperm samples due to several obstacles. Before the SVIA dataset, the lack of publicly available large-scale datasets that were suitable for training CASA systems was a major concern. Additionally, impurities, such as sperm clumping and background debris, have prevented accurate image analysis, which is required for a standalone routine clinical application [11,12,13]. Background debris or impurities in sperm image analysis include dead or deformed sperm [14]. Another consideration other than low accuracy is the need to obtain the analysis promptly with short inference time [15,16]. However, a fast inference time with low-accuracy performance would be meaningless. Determination of sperm from impurities can be considered one of the important factors in promoting CASA systems and having them even more widely implemented in clinical settings [10]. 

A recent dataset for sperm videos and image analysis called SVIA was collected and made publicly available. It consists of three subsets designed for different video and image analysis purposes. Subset-A is specific for object detection tasks, Subset-B is for image segmentation and tracking tasks, and Subset-C could be used for image classification tasks. This paper will focus on a classification task to clarify between impurities and sperm using Subset-C in the SVIA dataset. An impurity is a non-sperm object similar to sperm that can be bacteria, protein clumps, or bubbles, while the sperm class can contain a range of sperm morphological conditions including, normal, tapered, round, amorphous, pin or multi-nucleated heads. As of the writing of this paper, there has been no research that has classified the images in this SVIA Subset-C dataset.

### 1.3. Previous Studies

The HuSHeM and SCIAN datasets are the two most commonly used datasets for deep learning-based sperm classification [13,17,18,19,20]. The HuSHeM [13] dataset consists of 725 images, with only 216 of them containing sperm heads. In contrast, the SCIAN [20] dataset has 1854 sperm head images. A third sperm morphology dataset, SMIDS, compares three classes with a total of 3000 images recently available [21]. Previous research has mostly used convolutional neural network (CNN) [17,18,19], dictionary learning [13], or machine learning (ML) [20] models for classification. Research using a VGG16 transfer learning approach, called FT-VGG, achieved 94% accuracy on the HuSHeM dataset [18]. Another CNN-based study obtained 63% and 77% on the partial and full expert agreement on the SCIAN dataset, beating the previous state-of-the-art [18] method by an increase of 29% and 46%, respectively [19]. It also achieved 95.7% accuracy. Using a late (decision level) fusion architecture, a study by [21] achieved 90.87% accuracy on the SMIDS dataset. This particular research also investigated the model’s capability to replace rotation and cropping human intervention for automation purposes.

In addition to the SVIA dataset, several attempts at classification were made with the Subset-C dataset. In terms of accuracy, outstanding performers were the ImageNet pre-trained DenseNet121, InceptionV3, and Xception models. These models achieved 98.06%, 98.32%, and 98.43% on the accuracy metrics, respectively. Other pre-trained models attempted to classify the sperm images but obtained weaker results than the three mentioned above. In the research on sperm classification, the main problem associated with the low-performance scores is the lack of publicly available data, which was solved with the availability of the SVIA dataset. The works previously mentioned have shown their extraordinary abilities through machine learning because, although the shape of sperm is very subtle, it can be detected quickly and precisely with deep learning. The ability of this deep learning is indeed difficult to find in traditional doctors, but its results should not be used as the primary basis for medical decisions. It would be wiser to use it as supporting evidence. Thus, a major challenge is to create a deep learning model that minimizes this problem. Steps can be taken to create a deep learning model that can approach the actual value of truth. In this case, the authors propose a deep learning model that can beat benchmarks from previous works. By leveraging the large SVIA dataset, we propose a model that provides a more representative capability for sperm classification than existing models. This research could provide more accurate and generalizable models than existing ones while also performing more reliably than embryologists in mass analyses. Furthermore, this could propel efforts to standardize infertility treatment in clinics worldwide, facilitating its progress.

### 1.4. Proposed Method

In response to the shortcomings that were found in previous studies related to sperm morphology classification, this study was conducted to develop a deep-learning model that improved on those used in previous studies. We considered several gaps found in previous studies that could be mitigated in this research. Moreover, those gaps had not been addressed by previous studies. Therefore, based on those gaps, in this study, we added three main ideas for developing a sperm morphology classification, including transformer-based models, fusion techniques, and an autoencoder. The first one employs a transformer-based model that utilizes an attention mechanism well-known to capture global feature dependencies more efficiently than the recurrent neural network (RNN) or LSTM model. Since there were also concerns about the lack of local inductive biases for transformers used in vision tasks, a lightweight CNN-based MobileNetV3 was incorporated. Hence, both global and local features could be utilized for classification. Second, using an early fusion technique involving the feature maps generated from two separate models, a large feature map could be generated, and this would enrich the features from the small sperm images to improve the accuracy of classification. Third, using autoencoders within the architecture would alleviate the effect of unwanted noise in the sperm images without prior human intervention in the images, thus further improving classification predictions. Therefore, this model architecture would also remove the need for excessive human intervention and automate sperm morphology classification using a more robust method than previous studies have attempted.

This paper proposes a deep learning fusion architecture, called SwinMobile, that combines the shifted windows vision transformer (Swin) and MobileNetV3 to classify sperm and impurities in SVIA Subset-C. Both the Swin and MobileNetV3 could resolve the problems associated with sperm classification, as they leverage the ability of Swin transformers to capture long-range feature dependencies in images and the mobile-sized architecture optimization algorithms in the MobileNetV3 to maximize accuracy. Another variant of SwinMobile was also developed with an autoencoder (AE) architecture before the classification network. Due to AE’s ability to denoise images and extract only the necessary features, classification accuracy should be improved, as it would only focus on the important aspects of the image [22]. Essentially, it performs similarly to a PCA, whereby a PCA discovers the linear hyperplane, while an autoencoder unravels the hyperplane non-linearly.

The Swin model improves on the vision transformer (ViT), which lacks the inductive bias possessed by CNN, such as translational equivariance and locality, when trained on insufficient data [23,24]. Benefiting from the small images of sperm, Swin also adds a linear computational complexity to the image size by performing self-attention computation locally in each non-overlapping window with a fixed number of patches, and partitions the whole image [24]. Compared to sliding window-based transformers, Swin performs more than two times faster. It also outperforms other forms of vision transformers, ViT and DEiT, in terms of accuracy. On the other hand, MobileNetV3 is an improved version of MobileNetV2, with better accuracy and inference times [25]. It incorporates a platform-aware AutoML neural architecture search or NAS and NetAdapt algorithm that searches each layer’s optimal number of nodes. The resulting model would be optimized to provide maximum accuracy in short inference times for a given hardware platform.

With the combination of these mentioned architectures, the problem of low accuracy could be solved for CASA systems on the SVIA dataset. In addition, compression on the best-performing proposed model was attempted to increase the inference time and reduce our model size while maintaining similar performances. This is essential, as CASA systems need high accuracy and a relatively high inference speed. DenseNet121, InceptionV3, and Xception models with outstanding accuracy scores on the SVIA dataset formed the benchmark against our proposed models. Due to the differences in pre-processing and other preparatory methods not explicitly described in the paper, the three models were rerun on our environment and dataset with the same pre-processing method as our models to ensure a fair comparison. The trained models were evaluated using a three-fold and five-fold cross-validation technique on several performance metrics, namely F1-score and accuracy. The proposed models were also tested on other sperm morphology datasets, such as the HuSHem [13] and SMIDS [21], to assess their generalization ability. Comparison to the state-of-the-art models was based on F1-score and accuracy. As part of this study, we developed an automated feature fusion model to improve the classification accuracy of sperm morphology by leveraging the abilities of the various model architectures. With this approach, the advantages of the various architectures were expected to be reaped, such as the global long-range feature dependence of Transformers, local inductive convolutional bias and small size of MobileNet, and the AutoEncoder’s denoising ability. Hence, our proposed models could achieve better automatic classification performance than previous models while being mobile-friendly.

## 2. Materials and Methods

### 2.1. Dataset Information

**Figure 1:**
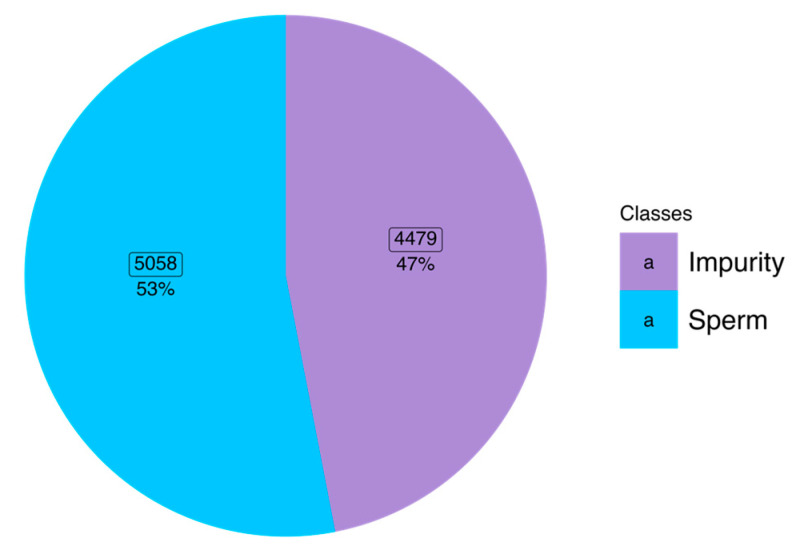
Distribution of classes within the balanced dataset.

All SVIA datasets were collected on 28 October 2022 [26] in compressed format. The dataset subsets were categorized into folders named Subset-A, Subset-B, and Subset-C. For the images to be compatible with further pre-processing and modeling, images in the subset-C folder had to be categorized into two class folders according to the names of each file. The file naming allowed differentiation between impurity and sperm images, denoted with an “I” and “S”, respectively. Hence, the two folder classes were “impurity” and “sperm”. The distribution of classes within the dataset was balanced following the amount used in the SVIA paper, with 5058 (53%) images belonging to the sperm class and 4479 (47%) images belonging to the impurity class [12]. Figure 1 shows the class distribution and percentage of each label. For performing k-fold cross-validation, the images were shuffled and randomly allocated into k partitions, resulting in train and test CSV files with the absolute file path and class label. A detailed explanation of the cross-validation method will be described in later sections.

**Figure 2:**
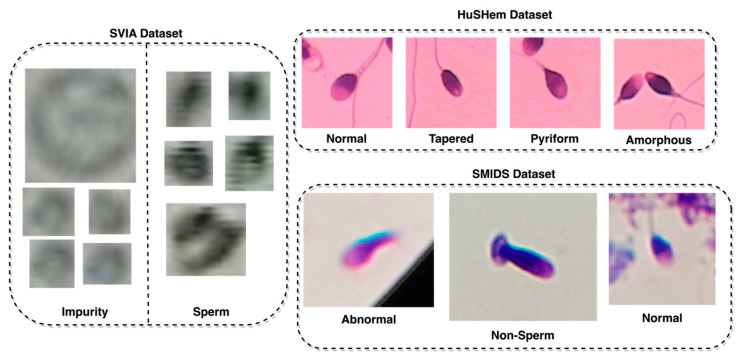
Sample images from the SVIA, HuSHem, and SMIDS datasets.

In order to assess the generalizability of the proposed model, the classification of images was also tested against other sperm datasets, such as the HuSHem [13] and SMIDS [21], using the proposed model. Both datasets differed from the classification task performed on the SVIA dataset, which only has two classes. The HuSheM dataset contains 216 publicly available images in RGB format with four classes, while the SMIDS contains 3000 images with three classes. Table 1 highlights the different properties across the three datasets. Another difference distinguishing the SVIA, HuSHem, and SMIDS is the RGB color space on the latter two datasets [13,21]. Lastly, there are differences in image size between the datasets. The HuSHem images are all 131 × 131, while the SMIDS has various sizes ranging from 122 × 122 to 259 × 201, and SVIA has sizes ranging from 2 × 2 to 150 × 172. The classes and number of images in the HuSHem are 54 Normal, 53 Tapered, 57 Pyriform, and 52 Amorphous sperm head images. The SMIDS contains 1005 Abnormal, 974 Non-Sperm, and 1021 Normal sperm head images. Several samples from each class in each dataset are displayed in Figure 2, with the SVIA images resized with an enlargement factor of 5 due to their small size.

### 2.2. Model Setup

**Figure 3:**
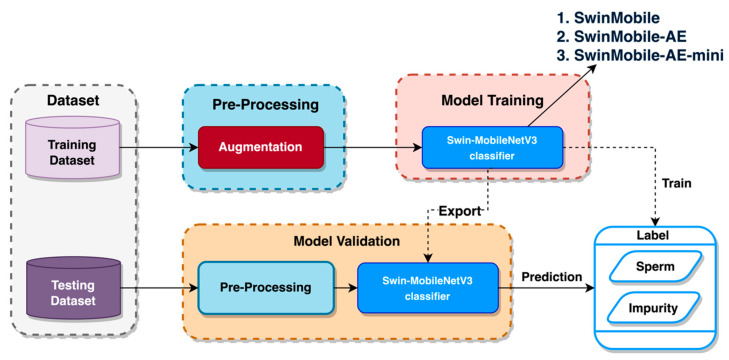
Experimental flow.

In this study, the datasets for training and testing were obtained following a cross-validation split stage. This stage will be discussed extensively in other sections. Pre-processing was performed on the training and testing datasets, consisting of several image augmentations. This augmentation aimed to increase the variation and number of images fed into the model, which would result in a performance boost [27,28]. Each of the augmentations applied to the images is discussed in the following section. The training dataset was used for the training stage to identify the labels between “sperm” and “impurity”. Once it completed its training, a final validation using the testing dataset was performed to predict the labels again. A visual representation of the experimental flow is shown in Figure 3. More detailed information on each experimental stage is provided in the corresponding sections below.

### 2.3. Data Pre-Processing

**Figure 4:**
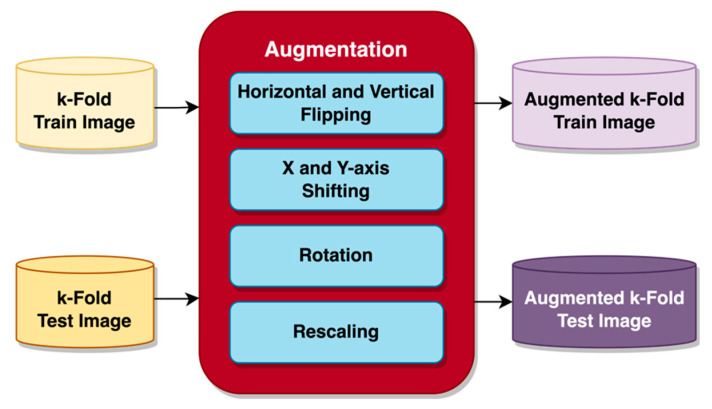
Augmentation on image data.

For the images to be suitable for the pre-trained models, each image was resized to a constant size of 224 × 224 with RGB channels during loading, then augmentation was applied. Image augmentation is frequently performed for image classification processes to increase the number of images in the dataset by generating new images not previously present in the original dataset [25]. In the case of limited datasets, augmentation would be particularly beneficial where achieving satisfactory training performance in some cases might otherwise not even be possible [26]. Applying such a method before feeding the image to the model significantly increases task performance [25]. An important issue associated with augmentation is the increased memory requirement [26]. Since the k-fold cross-validation prepares the data in the testing and training datasets, all of the images within the datasets are used without further splitting. Several augmentations to the input images, both for testing and training, were performed before feeding the models, as depicted in Figure 4. 

The images were augmented before usage by flipping, shifting, rotating, and rescaling the pixels. Image flipping was performed horizontally and vertically, and so was image shifting on the x-axis and y-axis using the height and width shift range function. With height and width shift range functions, the image is shifted by a percentage of its width. Height shift will shift the image between the y-axis, while width shift will perform shifts on the x-axis. A “nearest” fill mode was selected to replace the gaps left by image shifting with pixels closest to the remaining image border. A small rotation was also applied to further increase the variation of the images the model is trained on. Following the augmentation, the image’s pixels were rescaled from a range of 0 to 255 into a range of 0 to 1. Rescaling similarly affects normalizing pixels, speeds up the input process, and achieves convergence more quickly [29]. When applied, the resulting augmented image data are added to the dataset, thus increasing the volume of data within the dataset.

### 2.4. Swin Transformer

Transformers have become a capable model that extends beyond its initial domain of NLP into computer vision tasks and even tabular data processing [23,30]. The transformer-based model used for vision tasks, ViT, creates fix-sized patches of the image using patch embeddings, and another method called positional embedding retains information regarding the patch positions [23]. The resulting embedding vectors inserted into the Transformer encoder consist of the alternating multi-head self-attention (MSA) and multi-layer perceptron (MLP) or feed-forward. Layer normalization is added before each MSA and MLP layer and after the residual skip connection [23,31]. Although the performance is comparable to other state-of-the-art models, this model requires a large amount of training data and lacks inductive bias compared to CNN models [23]. Alternatively, the inputs for the model can be taken from feature maps formed by a CNN model [23,32].

**Figure 5:**
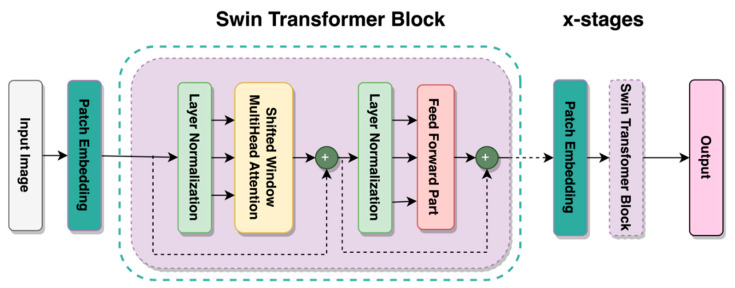
Architecture of Swin-T Transformer.

By improving the ViT using a shifted window mechanism, the Shifted Window Transformer or Swin Transformer was created [24]. This mechanism solves several issues associated with implementing transformers away from the text domain. A window partition is created on an image, where the MSA is computed on non-overlapping windows, and the MSA is used to establish relationships across windows. Another key quality of the Swin Transformer is the shifted window mechanism. It provides lower latency than sliding the window across the image while having comparable model performance. It can reintroduce inductive biases, locality, and translational equivariance, such as CNN architectures while utilizing relative positional biases [33]. This is implemented using the shifted window mechanism and local MSA computations on each of those windows, resulting in a more accurate representation of the image at a global scale [34]. All of these features of the Swin Transformer allow it to have high performance, low latency, and the potential to be used as a type of computer vision task backbone [24]. The variant of the Swin Transformer used in this study was the Swin Tiny or Swin-T, whose architecture is schematically represented in Figure 5. Other variants of the Swin Transformer build upon this basic structure.

According to Vaswani et al. [35], the transformer is entirely based on the attention mechanism, which requires inputs representing absolute position information. This model uses relative positional encoding to introduce bias [24,33]. In transformer-based models, a positioning embedding called relative positional encoding attempts to exploit pairwise, relative positional information through position embedding [36]. Keys and values are added to provide relative positional information as part of attention calculation instead of simply adding semantic embeddings.

### 2.5. MobileNetV3

**Figure 6:**
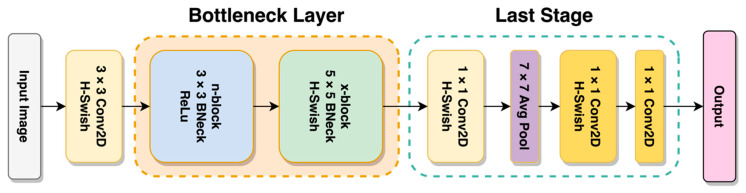
General architecture of MobileNetV3 Transformer.

In line with the goals of MobileNetV2, MobileNetV3 intends to achieve even greater accuracy and lower latency than previously achieved by state-of-the-art mobile platform models [25]. MnasNet, which predates MobileNetV3, was inspired by the architecture of MobileNetV2 and introduced attention modules in the bottleneck block based on the concept of squeeze and excitation before MobileNetV3. This third edition of the MobileNet model series uses a combination of neural architecture search (NAS) and is enriched further with the NetAdapt algorithm. Furthermore, several novel architectures have been implemented on top of these algorithms to make the model less resource-intensive, such as hard-swish activation functions and redesigning the last stage. As shown in Figure 6, the structure of MobileNetV3 consists of two main sections called the bottleneck layer, which is arranged invertedly, and the last stage block. It contains a modification to the V2 architecture that made the model even more accurate and less resource-intensive.

The V2 and V3 include the bottleneck (BN) architecture, described as a group of layers with smaller units in the middle layer than the other layers [37]. There are two structures to a bottleneck architecture: the encoding and decoding process [38]. Starting from the initial outer layer toward the middle layer, this can be considered an encoding process or compression. The compression that the BN architecture offers is nonlinear. Relevant information that passes through these layers is compacted, and redundancies are discarded [38]. The decoding process begins from the bottleneck layer and proceeds to the outermost layer, where the number of units (gradually) increases. Since the model complexity is reduced through the implementation of this architecture, the benefit of a reduction in overfitting models can also be obtained. Dimensionality reduction is also achievable through this architecture while providing superior generalization performance [39,40]. 

The inverted bottleneck structure and variants of MobileNetV2 are used in current models to expand their feature space to a higher degree by using 1 × 1 convolution [25]. Having rich features is essential for prediction. However, this comes at the price of latency and computation. We moved the layer past the final average pooling to reduce latency and maintain high-dimensional features. This final set of features is now computed at 1 × 1 spatial resolution instead of 7 × 7 spatial resolution. This method of design features low latency and cost-efficient computation. Specializing in each network block using platform-aware NAS platforms, MobileNetV3 can determine the overall network structure and create a neural network that optimizes quality, size, and latency [25,41]. It is then used in tandem with NetAdapt to search per layer for the number of filters for a particular layer by applying gradual changes to the filter and measuring resource consumption on each change [25,42]. In combination, these methods are complementary and can allow users to obtain optimized models suitable for a particular hardware platform [25].

### 2.6. AutoEncoder

The bottleneck structure used in the model design of the MobileNetV2 and V3 was built similarly to the AutoEncoder (AE) structure. Autoencoders are neural networks that learn to encode data without supervision [43]. Autoencoders consist of two parts: an encoder and a decoder. A hidden layer h generates a reduced feature representation based on the initial input x. The decoder reconstructs the original input from the encoder’s output by minimizing the loss function. An autoencoder reduces high-dimensional data to low-dimensional data. This makes the autoencoder especially useful for noise removal, feature extraction, and compression tasks.

AEs can be constructed using either fully connected layers or convolutional layers for the encoding and decoding section [44]. Several types of AEs can be developed with different applications, namely, the regularized AE group consisting of contractive, denoising, sparse AE, variational AE, and disentangled AE [22]. Their capability can also be applied in generative models, classification, clustering, anomaly detection, recommendation systems, and dimensionality reduction tasks, to name a few. In image tasks, AEs denoise images before compressing them to only meaningful representations and then reconstructing them back to images. Autoencoding unravels the hyperplane non-linearly, unlike PCA, which finds a linear hyperplane.

A dense AE architecture is developed using multiple deep fully connected layers instead of convolutional layers [44]. With the encoder nodes becoming smaller in the latent space and dense layer nodes gradually increasing for the decoder, it is assumed that the AE model is capable of compressing all of the input features into the latent space and then learning the relationships between the features before reconstructing them back into space size that similar to the input. 

### 2.7. Proposed Models

In this study, we put forward several models that could have the potential to outperform other models on the accuracy and inference time problems that are experienced in a CASA system. The SwinMobile and its AE and AE-mini variant combine two pre-trained model architectures and dense layers arranged in various architectures. The arrangement of the dense layer after the model fusion, as well as model parameters and size, play a relatively significant role in the accuracy and inference performance. In the following sections, each of the three models is described along with the parameters of each component.

#### 2.7.1. SwinMobile

**Figure 7:**
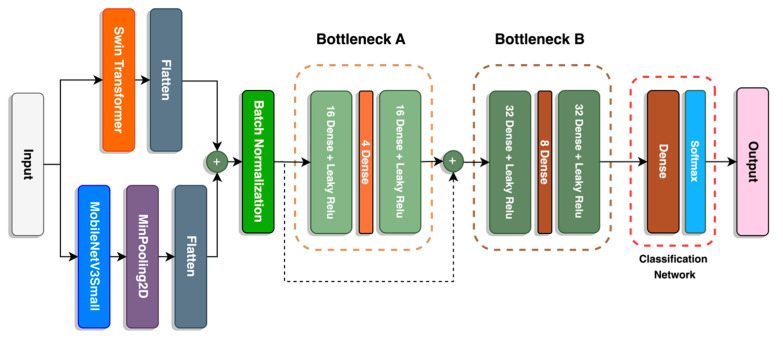
Architecture of SwinMobile.

A fusion between the Swin-T transformer and the MobileNetV3Small models forms the backbone of the image classification model. A schematic diagram of the model is available in Figure 7, and the parameters of each layer are described in Table 2. The output of both models is flattened before concatenation to match the output shape of both models. 

For the MobileNetV3 Small model, a MinPooling layer is applied before flattening. A MinPooling layer is suitable for images with lighter backgrounds, since it can extract darker pixels of the object [45]. Normalization per batch is applied after the first concatenation to reduce covariance shift, prevent model overfitting, and speed up model training [46]. It also has a regularizing effect that may replace dropout layers.

Bottleneck blocks are implemented into the model before the classification network. There are two bottleneck blocks, A and B, where the units in B are double that of A. Between the two bottleneck blocks is a concatenated skip connection that retains information from the previous layer and reuses it for the following layers. This form of skip connection is implemented in DenseNet [47] and Inception [48] networks. Bottleneck offers several benefits to the network, as it functions to reduce dimensionality and model complexity and prevents overfitting. A similar structure is used in both the V2 and V3 MobileNet models. Finally, the classification network completes the model with a dense layer with units equivalent to the number of classes with a “softmax” activation to convert the model’s raw output into a vector of probabilities that sum up to 1 [49].

#### 2.7.2. SwinMobile-AE

**Figure 8:**
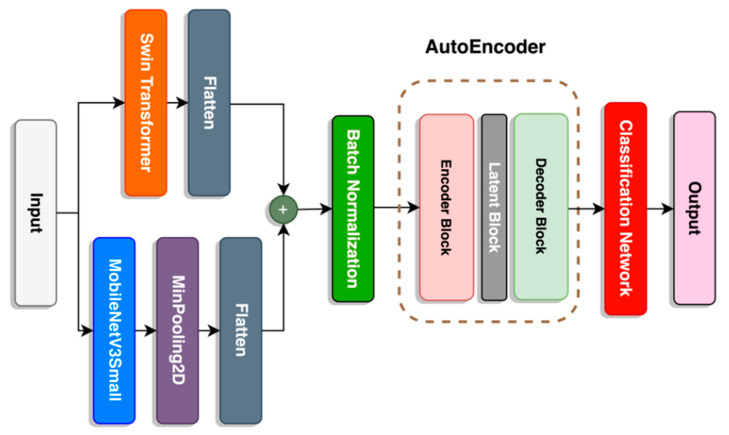
Architecture of SwinMobile-AE.

Due to relatively mediocre accuracy performance on the SwinMobile model, we explored using the AE architecture as the final stage before the classification network. As described in previous sections, the AE consists of an encoder, latent space, and decoder. This is applied in three-layer blocks with a gradual decrease in units toward the latent space, followed by a gradual increase in units, as seen in Figure 8. 

Unlike the encoder and decoder blocks, an activation function was not applied to the latent block. Although the latent block would linearly dissect the hyperplane, similarly to a PCA, it is transformed beforehand by the encoder block as it enters the latent block. However, this would not cause the whole AE structure to function like a PCA since the encoder and decoder blocks have a “Leaky Relu” activation function to transform the hyperplane non-linearly. Details on the parameters of the proposed SwinMobile-AE are described in Table 3. Adding the AE to the model significantly increased the model size and parameters.

#### 2.7.3. SwinMobile-AE-Mini

Since the AE variant of SwinMobile includes an AE stage before the classification network, the network is deeper. Therefore, it has more parameters and a larger model size than the non-AE variant. In order to reduce the model’s size and parameters, several modifications to the architecture were applied, such as the alpha, or depth multiplier, of the MobileNetV3Small and the layers as well as the units in the AE stage. The general structure of the model remains similar to that of the SwinMobile-AE, as described in Table 4.

The encoder block for this mini variant has only two layers compared to the three layers in the non-mini variant. It also has fewer units in each of the layers. The same reduction is applied to the latent block and the decoder block. Apart from the number of layers and units, the structure remains the same, with the encoder and decoder blocks equipped with the “Leaky Relu” activation function, while the latent block has “linear” activation. After the AE stage, the same classification network with units equal to the classes and a “softmax” activation is applied to obtain the output predictions.

## 3. Performance Evaluation

### 3.1. Model Training

The model was trained over 100 epochs, with a random iteration of three-fold and five-fold cross-validation. In order to optimize the weights, the stochastic gradient descent optimizer was used with a learning rate of 0.0001 as an initial learning rate for training. In this study, the cross-entropy loss function of categorical data was selected, along with a batch size of 64. The optimization algorithm is essential for practical DL model training, as it assigns appropriate weights and minimizes loss functions. This algorithm constantly modulates weights and learning rates on the network, improving accuracy and reducing overall loss. The proposed models applied a label smoothing cross-entropy loss function widely recognized by prior research, as shown in their classification application [50,51,52,53]. Label smoothing has a regularization effect on noisy labels, reduces the overconfidence of models, and improves classification [53,54]. Equation (2) can calculate the cross-entropy function, where y denotes the probability distribution of a prediction, and y’ refers to the actual probability prediction [53]. The label smoothing of the cross-entropy loss function is applied with a 0.1 smoothing factor. This would replace the $y_{i}^{’}$ in the regular cross-entropy function with $y_{i}^{LS}$ using Equation (1), where ∈ is the smoothing factor and *K* is the total number of prediction classes [53]. The complete cross-entropy loss function with label smoothing is shown in Equation (3).

(1) $$y_{i}^{LS}=y_{i}^{’}\left(1-\in \right)+\frac{\in }{K}\;\;\;\;$$

(2) $$L_{y^{\prime }}=-\underset{i}{\sum }y_{i}^{’}\log\left(y_{i}\right)\;\;$$

(3) $$L_{y^{\prime }}=-\underset{i}{\sum }\left(y_{i}^{’}\left(1-\in \right)+\frac{\in }{K}\right)\;\log\left(y_{i}\right)$$

Hyperparameters are indispensable in training a deep learning network [55]. They must be tailored to each architecture precisely to control the learning process. This approach aims to minimize the loss between the predicted output values and the actual output values. The loss metric, if it is smaller, correlates with better generalizability and higher accuracy. It is expected that when the instantiated loss is applied to the training system at the beginning, it will gradually decrease until it reaches a local or global optimum in line with the objective of the training process. This study training was performed using Python version 3 on a device with an AMD EPYC 7551P 32-Core@2.00 GHz, 32 GB of RAM, and accelerated with a GeForce RTX 3090 GPU.

### 3.2. Evaluation Scheme

**Figure 9:**
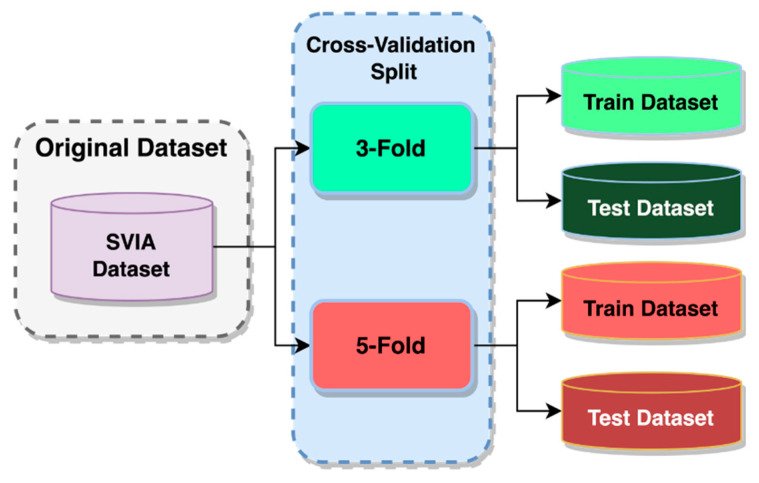
Cross-validation data split.

In order to validate our model further, k-fold cross-validation training and testing were incorporated into the study. It can validate multi-class classification tasks by distributing the dataset over several random groups [56]. It also provides insight into the true prediction error of models and for tuning model hyperparameters [57]. The data for training the model equaled k-1 folds, and the remaining fold was used for model testing [58]. As k partitions were created randomly, the proportion of sample classes between folds would likely differ. This benefited the training process to avoid overfitting a particular sample class and allowing better generalization on unseen data. This method of model validation is more frequently used due to its out-of-the-box nature, which enables usage for virtually any predictive model, unlike other methods such as BIC or AIC, which depend on a likelihood function or stochastic model [59]. The average k model performance metrics on k validation sets are considered cross-validated performance [57]. Two k-fold cross-validation methods were used to examine the developed models, a three-fold cross-validation and a five-fold cross-validation, as shown in Figure 9. 

In three-fold cross-validation, only 66.67% of the whole dataset will be available for training, while 80% of the data will be available in a five-fold cross-validation. The remaining data would be used for validation and testing purposes. Using two different k-folds, the model could be examined on different amounts of training data. That provided insight into how its performance is affected when fewer data are provided. Apart from that, the cross-validated performance of the models could be obtained. There was a high correlation between a higher number of folds and higher accuracy due to the larger training data available [60]. These cross-validated performances were then evaluated by comparing the benchmark models against the different variants of the proposed models. It would validate our results as having a better or worse performance. For the HuSHem and SMIDS datasets, due to their smaller dataset size, a split of training and testing data was applied, with 80% for training and 20% for testing, similar to a 5-fold cross-validation.

### 3.3. Evaluation Metrics

For the results of this study, the accuracy metric was used as the key performance indicator. Accuracy is a commonly used metric classification task [61,62]. It is useful in balanced classes where all the classes are equally important. However, it does not perform well on imbalanced data with varying importance. To calculate accuracy, divide the number of correct predictions, True Positives added with True Negatives, divided by the total number of predictions [63]. The equation to calculate the accuracy metric is shown in Equation (4), where *TP*, *TN*, *FP*, and *FN* refer to True Positive, True Negative, False Positive, and False Negative, respectively. Higher values for this metric are desirable over lower values.

(4) $$Accuracy=\frac{TP+TN}{TP+FP+TN+FN}$$

The metric called the *F*1*-score* can be computed from the harmonic mean between precision and recall [64,65]. This metric is useful when comparing different model performances and identifying true positives from false positives. *F*1*-score* is often used for imbalanced data due to its ability to consider the balance between the precision and recall of a classifier [66]. The best value for the *F*1*-score* is 1, and the worst is 0. In binary classification models, the *F*1*-score* can identify weak points of the classification model.

In contrast, a high macro-scale *F*1*-score* in multi-class classification would indicate better model classification ability across all classes [67]. Hence, it is also useful to apply when comparing different models for the same task. Equation (5) can be used to compute the *F*1*-score*.

(5) $$F1-Score=2\frac{Precision\;\times \;Recall}{Precision+Recall}\;\;$$

Precision refers to the relevancy of the result and penalizes False Positives, whereas recall describes the number of meaningful results returned by the model and penalizes False Negatives [68]. Both precision and recall can be computed using Equations (6) and (7), respectively.

(6) $$Precision=\frac{True\;Positive}{True\;Postive+False\;Positive}\;\;$$

(7) $$Recall=\frac{True\;Positive}{True\;Postive+False\;Negative}\;\;$$

## 4. Results

### 4.1. Comparison Parameters

In this study, we proposed several deep-learning classification models on three sperm head datasets using a combination of Swin, MobileNetV3, and AE architectures. The images from each dataset were separated into train and test k-fold fragments, as discussed in the previous section. They then underwent augmentation before feeding into the model. For the SVIA dataset, the classification task was performed on two classes: Sperm and Impurity. For the HuSHem dataset, this was performed on four classes: Normal, Pyriform, Tapered, and Amorphous, whereas the SMIDS data were classified into three classes: Normal, Abnormal, and Non-Sperm. Each dataset was evaluated using the scheme mentioned in the previous section based on the F1-score and accuracy. Since there were no benchmarks on the SVIA, it was compared to benchmark models. The parameters used for comparison were accuracy and F1-score, as these were the relevant performance metrics for comparison in previous research.

Additionally, training time, inference time, model size, and model parameters were compared only between our proposed models to provide insight into the effect of adding the AE architecture and compression into the mini version. However, since these models would be applied in the medical field, an accurate result was highly favored over inference speed. The results for the other two datasets were compared with the previous literature.

### 4.2. Overall Performance of Proposed Models

Across the three- and five-fold cross-validation, the cross-validated performance was gathered and summarized in Table 5 for accuracy, Table 6 for f1-score, and Table 7 for inference time across all three datasets. Bolded results show the best score. By altering the hyperparameters of the SwinMobile-AE architecture, the SwinMobile-AE-mini was developed as a compressed version. The best-performing model, in terms of accuracy, was the SwinMobile-AE. The compressed mini version still had similar performance despite having fewer parameters. Results on the SwinMobile-AE on the HuSHem and SMIDS were also relatively high. It achieved 97.6% and 91.65% classification accuracy, respectively. F1-scores on all three datasets are very similar to the accuracy results. Higher results are better for accuracy and f1-score, but lower values are favorable for inference time. The different number of test images available in each dataset could cause a significant disparity between inference time across different datasets.

### 4.3. SVIA Dataset Results

**Figure 10:**
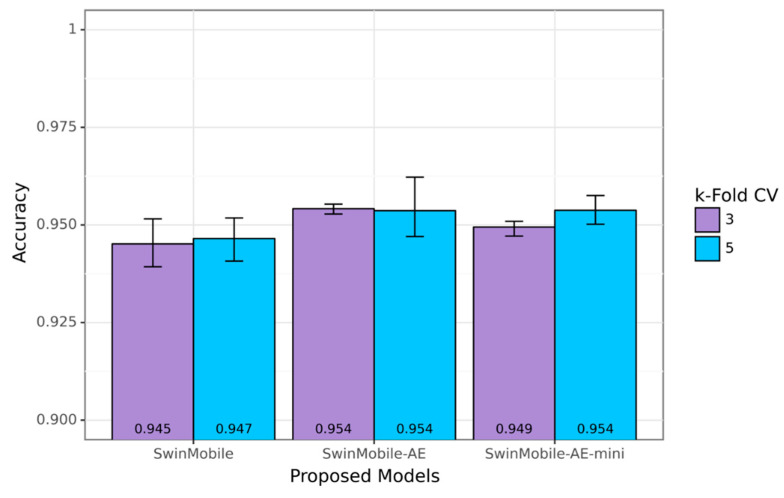
Accuracy performance range of proposed models on SVIA.

Since the SVIA dataset has the largest number of images compared to HuSHem and SMIDS, the range of accuracy performance across and between the different k-fold cross-validations was explored only on the SVIA. The proposed models achieved average accuracy results above 94.50%, with the higher end of the results exceeding 95% for all models on the SVIA. A bar graph with the lower and higher ends of each cross-validation result is shown in Figure 10. The average results are also shown at the bottom of the bars. Applying the AE architecture before the classifier network onto the base SwinMobile model architecture could increase the accuracy performance by over 0.5% at the cost of increased training time, inference time, model size, and model parameters. Among the proposed models, the best-performing model was SwinMobile-AE, with 95.39% accuracy and 95.39 F1-score. It was also found that higher k-fold values generated better model performance, as reported in the literature [60]. The base SwinMobile has a larger range of performance between the lower and upper ends. This was reduced by introducing the AE and lowering the hyperparameters in the model. However, a higher k-fold increased this range slightly. This may have been due to the increased model complexity, resulting in a more stable model. Consequently, a larger model is required for larger amounts of data.

The outcome of an attempt to reduce the impact of implementing AE into the SwinMobile could be observed with the SwinMobile-AE-mini. For this AE-mini variant of the SwinMobile, the training time, inference time, model size, and model parameters were successfully reduced compared to our highest-performing model. However, the compression did slightly reduce the performance of the model. Details of each value on the SVIA dataset are shown in Table 8. To understand the table better, a lower value for all of the metrics was desirable over higher values. Results in bold are considered the best result. Despite the reduction in model size and parameters, the AE-mini achieved similar average accuracy compared to the AE variant. The inference and training time of the AE-mini model were even lower than those of the SwinMobile, which had the smallest model size and number of parameters.

**Figure 11:**
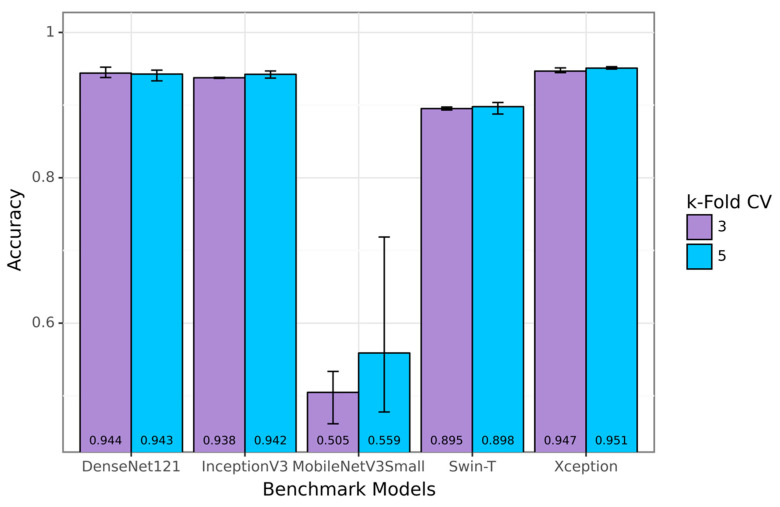
Accuracy results for benchmark models on SVIA.

Due to the large amount of data available in the SVIA dataset compared to other sperm classification datasets, some of the available pre-trained models could achieve high performance that exceeded 90% accuracy. Swin-T and MobileNetV3Small, used as the components for the proposed SwinMobile models, did not perform as well as other benchmark models, yielding averages of 89.69% and 53.85% accuracy, respectively. The highest-performing model on the benchmark was Xception, with 94.94% classification accuracy. Results for the benchmark models are shown in the bar graph in Figure 11, with the average results at the bottom of the bars. However, none of the benchmark models achieved over 95% classification accuracy when averaged across the two k-folds, whereas the proposed models achieved this feat. When the higher end of the cross-validation results was compared in each k-fold, all three proposed models outperformed the Xception model by at least 1%. Similarly to the proposed models, less complex models, such as the MobileNetV3, have a more comprehensive range of performance than more complex models. More data also seemed to introduce instability into performance when model complexity remained constant.

## 5. Discussion

To give better insight regarding the performance of the proposed SwinMobile model and its variants, the evaluation to validate our models was conducted by performing comparisons with other state-of-the-art models. The comparison was made to the benchmark models by implementing recent and existing deep learning models on the SVIA dataset due to the absence of literature that conducted classification research on the SVIA subset-C. However, comparisons were made against the findings of other works in the literature for the HuSHem and SMIDS datasets, which have been released for a longer period of time and have been the subjects of classification research. The proposed models succeeded in surpassing the existing models’ performance with quite some margin. Starting from the basic SwinMobile, they outperformed DenseNet121, InceptionV3, and both MobileNetV3Small and Swin-T with an average accuracy of 94.60%. However, they did not perform as well as the Xception model. The AE and AE-mini models were able to achieve an average of 95.39% and 95.21% average accuracy, respectively. Both outperformed all benchmark models, including the Xception model. These results are shown in Table 9.

The implementation of a machine learning technique based on support vector machine (SVM) was investigated by [67], while the employment of a deep learning scheme for the classification task was performed by [68,69]. Thus, we compared our proposed models with the implementations from [67,68] since the deep learning model of [69] was not constructed on the same datasets that we used. As shown in Table 10 and Table 11 for the HuSHem and SMIDS datasets, our best-scoring proposed model surpassed the performance of models in previous studies on the accuracy and F1-score metrics. On the HuSHem data, SwinMobile-AE achieved scores of 97.6%, which is almost a 2% increase over MC-HSH, and on the SMIDS data, it achieved a score of 91.65%. Several F1-score results were not available, as the results were directly taken from the respective literature. These results show that the SwinMobile-AE model has more robust classification ability compared to models in previous studies, as it not only achieved state-of-the-art accuracy scores, but also achieved this across three drastically differently sized datasets with varying numbers of classes and image color spaces. The dataset sizes were drastically different: the SMIDS had ~3000 images, the HuSHem less than 250, and the SVIA over 9000. The HuSHem and SMIDS datasets comprised four and three classes, respectively, while the SVIA had only two classes. Lastly, both the HuSHem and SMIDS were colored, whereas the SVIA was in grayscale. This is particularly novel, as previous studies had only tested their models’ robustness on the same color space. Due to the smaller size of both the HuSHem and SMIDS datasets compared to the SVIA, the batch size was modified to accommodate fewer data, with the HuSHem using a batch size of four and the SMIDS using a batch size of 32. The datasets were also split into training and testing datasets, similarly to the five-fold cross-validation, with 80% for training and 20% for testing. Other than that, the conditions for training and testing were identical.

Across all three datasets, the proposed SwinMobile-AE achieved better results than the state-of-the-art models. One of the main reasons for our models’ performance could be the combination of the Transformer (found in Swin-T) and CNN (found in MobileNetV3) models within the architecture. Previous studies mainly relied on CNN-based models. Apart from that, this feat was achieved due to the complementary strengths found in each of the modularities in our model architecture, including the Transformer in Swin-T, which can capture long-range feature dependencies and introduce parallel computations, the neural architecture search and platform-specific optimization from MobileNetV3, and the AE architecture that can parse through the noise in an image. Together, all of these combined strengths produced a robust and high-performing classification model. It was also observed that the classification models with less complexity were often more turbulent in their performance, while more complex models were more stable. With the increase in data, model stability can be maintained by increasing the model complexity. The proposed models, particularly SwinMobile-AE, had robust classification ability that enabled them to function with high classification results across three different datasets with different characteristics in terms of data size, number of classes, and color spaces. This study did not investigate using models trained on the SVIA dataset for transfer learning purposes. As the SVIA is currently the most significant sperm morphology public dataset, it can potentially be used for transfer learning into smaller datasets, such as the HuSHem, SMIDS, SCIAN, or other small sperm datasets. This would theoretically generate even better results. Further research on sperm morphology could investigate the use of SVIA in transfer learning. We also highly encourage investigating the effectiveness of our models on actual samples in clinical settings.

## 6. Conclusions

Extensive research has been conducted to identify sperm fertility to assist medical needs. Hence, deep learning architecture has been developed as a research subject to modernize and facilitate accurate decisions as part of state-of-the-art solutions. The final goal is to improve accuracy and achieve fast inference times, thus providing a fast-screening system. Therefore, we designed our architecture based on fusion deep learning with this situation in mind. Based on our research, our proposed architecture was shown to be a highly accurate method of classifying sperm. The SwinMobile-AE model approach that we propose achieves better results than state-of-the-art models with the selection of accuracy parameters for evaluations implemented in the clinical field, but we also explored other considerations to obtain fast analysis with the mini version of the model we propose. Our model outperformed the state-of-the-art Xception model on the SVIA dataset (95.4% vs. 94.9%). On the HuSHem dataset, our model surpassed MC-HSH [19] (97.6% vs. 95.7%). Further comparisons that strengthen the case that our models work well can be observed on the SMIDS dataset with the results from Ilhan et al. 2022 [21] (91.7% vs. 90.9%). Combining the Swin Transformer with the AE architecture supported this feat even for three datasets, relying solely on image input. The lessons learned show that increasing data can maintain model stability by increasing the model’s complexity. The proposed models—in particular, SwinMobile-AE—have powerful classification capabilities that enable them to function with high classification results across three different datasets, even with different characteristics regarding data size, number of classes, and color space. All of these findings highlight the potential for deep learning technology to create a modern sperm support system for the clinical setting by classifying sperm fertility to maintain human reproduction, because the evidence has been strengthened by existing evaluations, especially in terms of accuracy.

## Figures and Tables

**Table 1:** Properties of different sperm datasets.

Dataset	Image Colors	Classes	Dataset Size	Image Size
SVIA	Grayscale	2	>125,000	2 × 2 to 150 × 172
HuSHem	RGB	4	216	131 × 131
SMIDS	RGB	3	3000	122 × 122 to 259 × 201

**Table 2:** Parameters of SwinMobile-AE-mini.

Layer	Parameter	Value
Swin-T Transformer	A variant, Input Size	Imagenet 1K Pre-Trained, 224
MobileNetV3Small	Weights, alpha	Imagenet 1K Pre-Trained, 1.0
	MinPooling2D	
	Flatten	
	Batch Normalization	
Encoder Block	1st Dense, Activation	16, ‘leaky Relu’
	2nd Dense, Activation	4, ‘leaky Relu’
Latent Block	Dense, Activation	16, ‘leaky Relu’
Decoder Block	1st Dense, Activation	32, ‘leaky Relu’
	2nd Dense, Activation	8, ‘leaky Relu’
Classification Network	Dense, Activation	32, ‘leaky Relu’

**Table 3:** Parameters of SwinMobile-AE.

Layer	Parameter	Value
Swin-T Transformer	Variant, Input Size	Imagenet 1K Pre-Trained, 224
MobileNetV3Small	Weights, alpha	Imagenet 1K Pre-Trained, 1.0
	MinPooling2D	
	Flatten	
	Batch Normalization	
Encoder Block	1st Dense, Activation	512 ‘leaky Relu’
	2nd Dense, Activation	128, ‘leaky Relu’
	3rd Dense, Activation	128, ‘leaky Relu’
Latent Block	1st Dense, Activation	16, ‘linear’
	2nd Dense, Activation	8, ‘linear’
	3rd Dense, Activation	16, ‘linear’
Decoder Block	1st Dense, Activation	32, ‘leaky Relu’
	2nd Dense, Activation	32, ‘leaky Relu’
	3rd Dense, Activation	128, ‘leaky Relu’
Classification Network	Dense, Activation	2, ‘softmax’

**Table 4:** Parameters of SwinMobile-AE-mini.

Layer	Parameter	Value
Swin-T Transformer	A variant, Input Size	Imagenet 1K Pre-Trained, 224
MobileNetV3Small	Weights, alpha	Imagenet 1K Pre-Trained, 0.75
	MinPooling2D	
	Flatten	
	Batch Normalization	
Encoder Block	1st Dense, Activation	256 ‘leaky Relu’
	2nd Dense, Activation	64, ‘leaky Relu’
Latent Block	Dense, Activation	8, ‘linear’
Decoder Block	1st Dense, Activation	16, ‘leaky Relu’
	2nd Dense, Activation	64, ‘leaky Relu’
Classification Network	Dense, Activation	2, ‘softmax’

**Table 5:** Accuracy performance of the proposed models on SVIA, HuSHem, and SMIDS.

Model	SVIA	HuSHem	SMIDS
SwinMobile	94.6%	87.8%	88.8%
SwinMobile-AE	95.4%	97.6%	91.7%
SwinMobile-AE-mini	95.2%	92.7%	90.7%

**Table 6:** F1-score performance of the proposed models on SVIA, HuSHem, and SMIDS.

Model	SVIA	HuSHem	SMIDS
SwinMobile	94.6	88.3	88.8
SwinMobile-AE	95.4	97.6	91.6
SwinMobile-AE-mini	95.2	92.6	90.6

**Table 7:** Inference time (in seconds) of the proposed models on SVIA, HuSHem, and SMIDS.

Model	SVIA	HuSHem	SMIDS
SwinMobile	30.1	1.3	7.3
SwinMobile-AE	30.2	1.2	7.2
SwinMobile-AE-mini	29.7	1.5	7.2

**Table 8:** Metrics of proposed models on SVIA.

Model	Avg. Training Time (min)	Avg. Inference Time (s)	Model Size	Model Parameters
SwinMobile	173.37	30.09	**112.96**	**29.22 M**
SwinMobile-AE	173.09	30.22	130.99	33.95 M
SwinMobile-AE-mini	**170.78**	**29.69**	117.31	30.37 M

**Table 9:** Performance comparison of the proposed models against benchmark models on the SVIA dataset.

	Model	Avg. Accuracy	Avg. F1-Score
Benchmark Models	DenseNet121	94.3%	94.3
	InceptionV3	94.1%	94.1
	MobileNetV3Small	53.9%	39.7
	Swin-T	89.7%	89.7
	Xception	94.9%	94.9
Proposed Models	SwinMobile	94.6%	94.6
	SwinMobile-AE	**95.4%**	**95.4**
	SwinMobile-AE-mini	95.2%	95.2

**Table 10:** Performance comparison of the proposed models against models in previous studies on the HuSHem dataset.

Model	Accuracy	F1-Score
CE-SVM [69]	78.5%	78.9
Yüzkat et al. 2021 [70]	85.2%	-
SwinMobile **(Our Model)**	87.8%	88.3
Ilhan et al. 2022 [21]	92.1%	-
APDL [13]	92.2%	92.9
SwinMobile-AE-mini **(Our Model)**	92.7%	92.6
FT-VGG [18]	94.0%	94.1
MC-HSH [19]	95.7%	95.5
SwinMobile-AE **(Our Model)**	**97.6%**	**97.6**

**Table 11:** Performance comparison of the proposed models against models in previous studies on the SMIDS dataset.

Model	Accuracy	F1-Score
SwinMobile (Our Model)	88.8%	88.8
SwinMobile-AE-mini **(Our Model)**	90.7%	90.6
Yüzkat et al. 2021 [70]	90.7%	-
Ilhan et al. 2022 [21]	90.9%	-
SwinMobile-AE **(Our Model)**	**91.7%**	**91.6**

## Data Availability

The data presented in this study are openly available. SVIA https://doi.org/10.6084/m9.figshare.15074253.v1, acessed on 20 November 2022; HuSHem https://doi.org/10.17632/tt3yj2pf38.1, acessed on 25 November 2022; and SSMIDS https://doi.org/10.17632/6xvdhc9fyb.1, acessed on 20 November 2022.
